# Outcrop coordinates and stratigraphic dip data of the Tambach Formation in the Tambach-Dietharz sedimentary basin, Central Germany

**DOI:** 10.1016/j.dib.2022.108368

**Published:** 2022-06-09

**Authors:** Frank Scholze

**Affiliations:** Friedrich Schiller University Jena, Institute for Geosciences, Burgweg 11, 07749 Jena, Germany

**Keywords:** Dipping azimuth, Dipping angle, Mapping, Rotliegend, Thuringia, Thuringian Forest mountains

## Abstract

Coordinates of surface outcrops in the Tambach Formation (Rotliegend Group, early Permian) as well as stratigraphic dip azimuth and dip angle values were measured newly during recent mapping activities in the Tambach-Dietharz sedimentary basin. In total, 304 localities of the Tambach Formation were measured. The dataset includes stratigraphic dip measurements of in-situ beds from all subunits of the Tambach Formation (i.e., Bielstein Conglomerate Member, Tambach Sandstone Member, Finsterbergen Conglomerate Member). The sedimentary rocks are composed of fine- to coarse-grained, reddish coloured siliciclastics resulting from alluvial and fluvial sediment transportation and deposition in an intracontinental palaeoenvironment. Longitude and latitude coordinates of the outcrop sections were measured with a handheld GPS device. Values of both the dipping azimuth and the dipping angles were measured with a geologic compass. The dataset has reuse potential for both cataloguing surface outcrops of the Tambach Formation and geologic mapping in the Tambach-Dietharz sedimentary basin. Additionally, the dipping azimuth and the dipping angle data can be reused for analysing the sedimentary basin geometry and digital geological modelling (2D, 3D) of the Tambach Formation.

## Specifications Table

**Table:** 

Subject	Stratigraphy
Specific subject area	Field measurements of outcrop coordinates, stratigraphic dipping azimuth, and stratigraphic dipping angle
Type of data	Table
How data were acquired	Surface outcrops of the Tambach Formation were mapped in the Tambach-Dietharz sedimentary basin. Coordinates of the local outcrop sections were measured with a GPS device. Both stratigraphic dipping azimuth and stratigraphic dipping angle of in-situ beds were measured with a geological compass. All measured values were noted physically by the author in a paper field book.
Data format	Raw Analysed
Parameters for data collection	All outcrop sections are located in the Tambach-Dietharz sedimentary basin. Sections either include active and abandoned quarries, road and trail cuts, roadside gullies, steep river banks or natural cliff exposures. Data collection considered that all values measured on-site (i.e., coordinates, stratigraphic dipping azimuth, stratigraphic dipping angle) belong lithostratigraphically to the Tambach Formation (Rotliegend Group, early Permian).
Description of data collection	The Tambach-Dietharz basin was explored in search of surface outcrops by the author in 2020–2022. In all found outcrops of the Tambach Formation, the coordinates of the respective outcrops, dips (i.e., azimuth, angle) of strata were measured. The measured values were recorded in a field book. Afterwards, the data were processed electronically utilizing a Geographic Information System (i.e., using the free software qGIS). A geographic basemap in the qGIS-software allowed both analysing the precision of measured GPS coordinates and correcting erroneous GPS measurements if necessary.
Data source location	Region: Tambach-Dietharz sedimentary basin (within the Thuringian Forest mountains) Country: Free State of Thuringia (central Germany) Latitude and longitude of selected outcrop sections in the study area: N50.84819400, E10.59852800 (northernmost section); N50.81494400, E10.66736100 (easternmost section); N50.76111100, E10.63086100 (southernmost section); N50.82377800, E10.57544400 (westernmost section).
Data accessibility	Repository name: figshare Data identification number: 10.6084/m9.figshare.19732744 Direct URL to data: https://doi.org/10.6084/m9.figshare.19732744

## Value of the Data

• The measured values of both dipping azimuth and dipping angle of the sedimentary beds are necessary for a future study of the geometry of the Tambach-Dietharz basin and the internal stratigraphy of this basin. This is important for developing a better understanding of the real geographic and stratigraphic dimensions of this sedimentary basin, its basin fill history, the basin exhumation, and the local structural geology (e.g., occurrences of faults and flexures).
• Prior to the present work, values of the dipping azimuth and the dipping angle for stratigraphic beds in the Tambach-Dietharz basin had been incomplete in state-official geologic maps (scale 1:25.000). The new data reported herein is useful for any geoscientist who is interested in the regional geology (e.g., outcrop sections, dip of stratigraphic beds) of the Tambach-Dietharz basin.
• This newly generated information can be reused for cataloguing and georeferencing surface outcrops of the Tambach Formation. The stratigraphic dipping data can be reused as a component for digital geological 3D-modelling.

## 1 Data Description

All primary data are listed in a single table that is saved as .txt-file and provided in the referenced Data Repository. This table is composed of eight columns, which are separated by semicolons. The first column includes a successively numbered ID for each outcrop. The second column contains the stratigraphic dipping data (combined “dipping azimuth/dipping angle” notation). The third column yields the abbreviation for the respective lithostratigraphic member of the Tambach Formation, i.e. roTc1 (=Bielstein Conglomerate Member, also called informally “Lower Conglomerate of the Tambach Formation”), roTs (=Tambach Sandstone Member), and roTc2 (=Finsterbergen Conglomerate Member, also called informally “Upper Conglomerate of the Tambach Formation”). The assignment to the respective members follows the state-official geologic map (scale 1:25.000) that is published as “Geologische Karte von Thüringen 1:25.000” by the geological survey of the Free State of Thuringia (i.e., “GK25” sheets no. 5129/Waltershausen [1], no. 5229/Tambach-Dietharz [2], no. 5130/Ohrdruf [3]). These lithostratigraphic members of the Tambach Formation were determined during fieldwork following distinct lithostratigraphic criteria that had been defined officially by the German “Subkommission für Perm–Trias-Stratigraphie” [4]. The fourth column includes the date when the local measurement values (i.e., dipping azimuth, dipping angle, GPS coordinates) were generated on-site during fieldwork. The fifth and sixth columns include outcrop latitude and longitude coordinates, respectively.

Additionally, the .txt-file in the referenced Data Repository yields in its seventh and eighth columns dipping azimuth and dipping angle values, respectively. Their listing in two separate columns intends supporting a reuse of the dip data, e.g., facilitating their import into geological 3D modelling software. All values were obtained from surface outcrops of the Tambach Formation [5], [6], [7], [8], [9] in the Tambach-Dietharz sedimentary basin [10], [11], [12], [13], [14].

## 2 Experimental Design, Materials and Methods

All field measurements were performed newly by the author during mapping in November 2020 to April 2022. Measurements of both GPS and stratigraphic dipping (azimuth, angle) were made directly on-site in local surface outcrops of the Tambach Formation in the Tambach-Dietharz sedimentary basin. Coordinate values were measured using a Garmin eTrex GPS device. The stratigraphic dipping azimuth and angle of in-situ beds were measured using a Breithaupt Gekom-N Pro geological compass. Measured values of the dipping azimuth and dipping angle were noted on-site in a field book, using a notation as follows: dipping azimuth (varying between 000° and 360°)/dipping angle (varying between 00°–90°); GPS coordinates were noted in the field book as well (notation: Degree° Minutes' Seconds''), using the WGS-84 datum. The estimated precision for visual reading of the geological compass measurement values is about ±1° and ±2° for the dipping azimuth and dipping angle, respectively, based on the author's field observations. The precision of the handheld device for the GPS coordinate measurements varied from ±3 m (measuring of outcrop sections under the free sky) to ±30 m (measuring directly in front of steep cliff outcrops).

All measured values (i.e., GPS coordinates, dipping azimuth, dipping angle) were entered into the program qGIS by hand typed [15]. Plotting the geographic positions of outcrops on the georeferenced qGIS basemap allowed analysing the measured GPS values for their correctness. Additionally, further data (i.e., date of the respective field measurement, lithostratigraphic assignment) were added to the qGIS-project. Finally, the dataset was exported from the qGIS-project as a txt-file that can be accessed utilizing the software Windows Notepad by Microsoft. This file includes for each measured outcrop section the following data (each separated by a semicolon): ID-number; the stratigraphic dip values, notation as “dipping azimuth/dipping angle”; the lithostratigraphic assignment (roTc1, roTs, roTc2); the date of the fieldwork, noted as “dd.mm.yyyy”; coordinate of the latitude in Decimal Degree (Lat°); coordinate of the longitude in Decimal Degree (Lon°); the dipping azimuth value; the dipping angle value.

## Ethics Statement

There is no reportable ethics statement in relation to this article, because this work does not involve the use of animal or human subjects.

## CRediT Author Statement

**Frank Scholze:** Investigation, Methodology, Data curation, Software, Writing – original draft, Writing – review & editing.

## Declaration of Competing Interest

The author declares that he has no known competing financial interests or personal relationships which have or could be perceived to have influenced the work reported in this article.

## Data Availability

Outcrop coordinates and dip values for the Tambach Formation in the Tambach-Dietharz sedimentary basin (Original data) (figshare).
